# Volumetric Variability of the Ventromedial Prefrontal Cortex Reflects the Propensity for Engaging in High-Stakes Gambling Behavior

**DOI:** 10.3390/brainsci12111460

**Published:** 2022-10-28

**Authors:** Kyuli Lee, Nayoung Kim, Eun-Joo Jeong, Min-Suk Kang, M. Justin Kim

**Affiliations:** 1 Department of Psychology, Sungkyunkwan University, Seoul 03063, Korea; 2 Center for Neuroscience Imaging Research, Institute for Basic Science, Suwon 16419, Korea

**Keywords:** risk, decision-making, vmPFC, voxel-based morphometry, MRI

## Abstract

The human ventromedial prefrontal cortex (vmPFC) has been traditionally associated with decision-making under risk. Neuroimaging studies of such decision-making processes have largely focused on patients with vmPFC lesions or pathological gambling behavior, leading to a relative paucity of work focusing on the structural variability of the vmPFC in healthy individuals. To address this, we developed a decision-making task that allowed healthy players to choose to participate in either low stakes or high-stakes gambling on a trial-by-trial basis, and computed a metric that indexes the propensity for engaging in gambles with greater potential payoffs. We leveraged voxel-based morphometric analyses to examine the association between prefrontal gray matter volume and individual differences in the propensity for seeking high-risk/high-reward situations. Our analyses showed that vmPFC gray matter volume was inversely correlated with an increased tendency for engaging in high-stakes gambling. These results converge with findings from functional neuroimaging and brain lesion studies of vmPFC, and further extend them to show that normative variability in brain structure could also underpin risk-taking behavior.

## 1. Introduction

Neuroeconomics research has identified the ventromedial prefrontal cortex (vmPFC) and orbitofrontal cortex (OFC) as the primary brain regions underpinning human decision-making behavior [1]. By extension, alterations in vmPFC/OFC have been often targeted by studies that sought to elucidate the neurocognitive mechanisms for pathological choice behaviors, with gambling being the most notable example [2]. Patients with vmPFC/OFC lesions prefer making decisions that lead to an immediate payoff in gambling, even when that immediate reward choice, which gradually changes to punishment, results in an overall loss [3]. To this end, the Iowa Gambling Task (IGT) has been widely used to examine changes in decision-making behavior [4]. Other tasks, such as the Cambridge Gamble Task [5] and the Cups Task [6], have also been developed. Largely consistent with the findings from IGT, patients with right frontal lesions made more detrimental choices during both tasks [7,8].

Based on these seminal findings from the lesion literature, neural bases of decision-making behavior were further explored via non-invasive neuroimaging methods, mostly notably magnetic resonance imaging (MRI) techniques. To date, neuroimaging studies of decision-making behavior have largely focused on elucidating its underlying brain function. For example, Rogalsky and colleagues [9] used functional MRI (fMRI) to show specific patterns of vmPFC activity during IGT. Increased activity of the right vmPFC was observed during gambling, whereas left vmPFC activity correlated with IGT performance [9]. Another study demonstrated greater vmPFC activity in high-risk rather than low-risk decisions [10].

As fMRI has shed light on our understanding of the neural mechanisms of decision-making, studies utilizing structural brain imaging techniques have also provided further insight into the role of the vmPFC in gambling behavior. High-resolution structural MRI can be utilized in various ways, one of which is to extract volumetric information of gray matter tissue in vivo. Such methods have been used to examine gray matter differences associated with gambling disorder [11,12]. Pathological gamblers, for example, exhibited smaller vmPFC/OFC gray matter volumes than healthy controls [13,14].

Structural MRI-derived metrics are usually highly reliable, and thus particularly suited for research into individual differences [15]. By leveraging this strength of structural MRI, we designed a study that attempted to link individual differences in the patterns of decision-making behavior with the variability in gray matter volume in healthy individuals. As earlier lesion studies have consistently highlighted deficits in decision-making behavior and reduced vmPFC/OFC gray matter volume was associated with pathological gambling, we sought to expand these findings by hypothesizing that the individual differences in vmPFC/OFC volume within the normal range might reflect their propensity to engage in gambling. Specifically, based on the tendency of vmPFC/OFC patients to prioritize reward, we predicted that individuals with a relatively smaller vmPFC/OFC volume would be more inclined to participate in gambling when greater stakes and payoffs are involved. Of particular importance, we sought to avoid relying on an experimental design in which the participants were forced to take part in a gambling situation (i.e., participants’ agency was taken away by not providing an option to not gamble), as is the case for many previously published decision-making tasks [16,17]. In the real world, people show individual differences in the propensity to engage in a gambling situation based on the stakes—some may be attracted to high-stakes gambling with greater potential payoffs, while others may actively avoid such risks. Therefore, to reflect real-world situations, we designed a task in which the participants can check the stakes during each trial before choosing whether to gamble or not. This allowed us to leverage each participant’s trial-by-trial behavioral responses to compute an individual differences metric that indexes the propensity to engage in gambling. Here, expanding upon the findings from lesion and pathological gambling studies, we present evidence that the volumetric variability of the vmPFC/OFC within the normal range is associated with the behavioral tendency to engage in high-stakes gambling.

## 2. Materials and Methods

### 2.1. Participants

**Figure 1:**
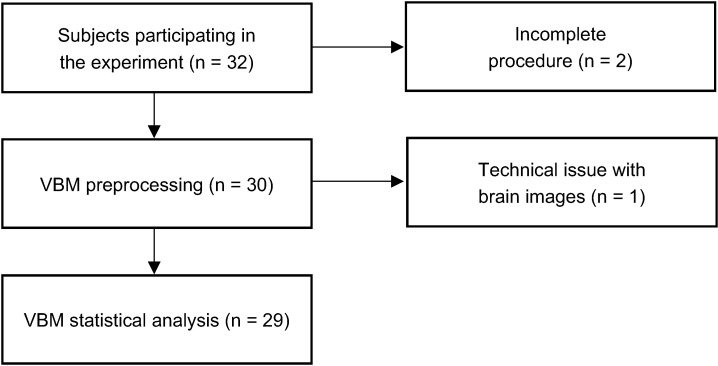
Summary of the study procedure.

A total of 32 participants (12 females; age range: 18–29 years; mean age: 21.5 years) were recruited for this study, which was a part of a larger research project. All participants were free of self-reported neurological or psychiatric disorders with normal or corrected-to-normal vision. They were paid KRW 20,000 per hour for 2 h of participation and additionally, KRW 10,000 were given as an incentive for those who completed all the procedures. Ethical approval was obtained from the Institutional Review Board (IRB) of Sungkyunkwan University. All participants gave informed consent, and all deceptions were debriefed at the end of each experiment. Out of the initial 32, 3 participants were excluded from the analysis due to the participants not being able to complete the experimental procedure (*n* = 2) and a technical issue with the anatomical brain images (*n* = 1). Thus, data from a total of 29 participants (11 females; age range: 18–27 years; mean age: 21.5 years) were included in the final analysis (Figure 1).

### 2.2. Experimental Task

**Figure 2:**
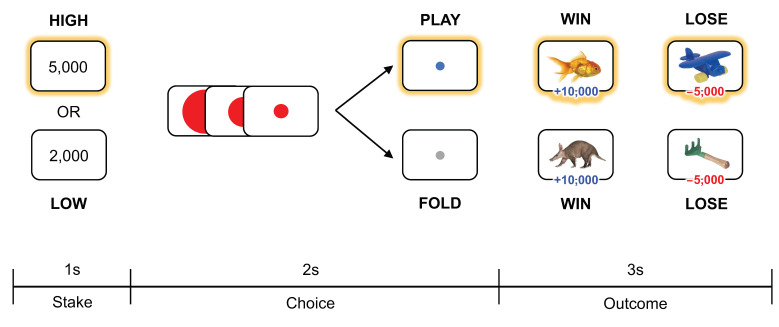
Task structure of the card-guessing task. Participants were first provided the amount of the ante for a given trial, which indicated whether the stakes were high or low. Then, within a 2-s window, participants chose whether to play or to fold. For example, after recognizing that the stakes are high, participants may choose to engage in gambling, which results in either winning or losing a set amount of money determined by the ante (highlighted in yellow).

All participants completed a card-guessing task in the MRI scanner (Figure 2). In this task, participants were instructed to make a guess about the category of a given card that was a picture of an animal or an inanimate object that was either associated with a win or a loss. Color images of animals and objects were collected from a previous study [18] and Google image search. Each trial started with the presentation of the value of the token required to gamble (KRW 2000 or KRW 5000, equating to about USD 1.50 and USD 4.00 each, corresponding to low or high stakes). Then, as the reverse side of a single card was presented on the computer screen, participants were asked to choose to place their bet that was of equal value to the presented token on the card, or to opt out of the game. With the exception of the amount of ante required to gamble (high vs. low stakes), participants were not provided with any cues that could guide their decision to engage in or opt out of the game. As such, participants solely relied on their beliefs and expectations for a given card on each trial. Based on their guesses, participants chose to play or pass the game with a button press within a two-second window. During this choice phase, a red circle was presented at the center and slightly shrunk over time and disappeared within 2 s. After the choice phase, the face of the card was displayed on the computer screen for 3 s, regardless of their choice to play or not. When the participants played the game and won, they received double the amount of the ante (KRW 4000 for low stakes, KRW 10,000 for high stakes); when they lost, the amount equivalent to the ante was taken away. The task consisted of 168 trials (84 unique animals and 84 unique objects). The images were shown to participants without replacement; as such, the winning probability was initially 50:50 but updated on a trial-by-trial basis, depending on the category of the image from the previous trial. Trials were grouped into 4 blocks (2 high stakes blocks and 2 low stakes blocks). As there were equal number of images per category, the overall wining probability across each block was set at 50%. The order of the blocks was randomized across participants. Psychophysics toolbox running on MATLAB was used to present the task and acquire the behavioral data.

Prior to the experiment, we explicitly informed the participants that they would receive the actual amount of money that was randomly drawn from the outcomes of their choice in the task at the end of the scanning session. Although participants were informed that the incentive would be random, we gave the maximum amount of money the game could yield as an incentive to all the participants who completed the task. This deception was fully debriefed at the end of the experiment.

### 2.3. Behavioral Index

Based on the behavioral responses recorded for all 168 trials in the card-guessing task, each individual’s propensity for engaging in high- vs. low-stakes gambling trials was computed using the following formula: ((# of trials accepted for high-stakes gambling)–(# of trials accepted for low-stakes gambling)). This behavioral index was used to assess the associations with brain volumetric measures. Control analyses were performed using (1) (# of trials accepted for high-stakes gambling) and (2) (# of trials accepted for high- or low-stakes gambling) instead of the aforementioned behavioral index to confirm that vmPFC/OFC volume does not reflect the simple frequency in which an individual engages in high-stakes gambling behavior, or in any gambling behavior regardless of the stakes and payoffs.

### 2.4. Image Acquisition

All MRI data were collected at the Center for Neuroscience Imaging Research at Sungkyunkwan University, using a 64-channel head coil with a 3.0 Tesla Siemens Prisma scanner. High-resolution anatomical T1-weighted images were acquired using a magnetization prepared rapid acquisition gradient echo (MPRAGE) pulse sequence with 192 sagittal slices (TR = 2300 ms, TE = 2.28 ms, FOV = 256 mm, flip angle = 8°, voxel size = 1 mm × 1 mm × 1 mm).

### 2.5. Voxel-Based Morphometry

#### 2.5.1. Preprocessing

T1-weighted images from all 29 participants were submitted to an optimized voxel-based morphometry (VBM) [19] data analysis pipeline, using FSL-VBM [20] implemented within FSL [21]. First, T1-weighted images were brain-extracted using the Brain Extraction Tool (BET). Brain-extracted images were segmented into three types of tissues of the brain, which are gray matter, white matter, and cerebrospinal fluid. Next, the segmented gray matter images were registered to MNI-152 standard space using nonlinear registration [22]. The standardized gray matter images were averaged and flipped along the x-axis to create a study-specific gray matter template. All native gray-matter images were then nonlinearly re-registered to a study-specific template. Next, the re-registered images were modulated using the Jacobian determinants derived from the nonlinear registration procedure to correct for the expansion or contraction due to the nonlinear component of the spatial transformation. Finally, the modulated segmented images were then smoothed with a Gaussian kernel of a full-width half-maximum of 8 mm.

#### 2.5.2. Statistical Analysis

To test for correlations between the behavioral propensities for engaging in high-stakes gambling and PFC gray matter volume on a voxel-by-voxel basis, a general linear model (GLM) was generated at the group level. As per our a priori hypothesis, we imposed a PFC region of interest (ROI) for the GLM analysis. PFC ROIs were constructed by combining all prefrontal regions as defined by the Harvard–Oxford cortical structural atlas. Permutation-based non-parametric testing (5000 permutations) was performed to determine a statistical threshold of family-wise error corrected *p* < 0.05, using the threshold-free cluster enhancement (TFCE) method [23].

## 3. Results

### 3.1. Gambling Behavior

On average, participants chose to engage in gambling activity on 55.2% of the trials (92.8 ± 21 trials). Overall differences in engaging in high-stakes gambling (44.9 ± 15.4 trials) and low-stakes gambling (47.8 ± 10.7 trials) were not statistically significant (*t*(28) = 0.97, *p* = 0.34). The behavioral index that measures the propensity for engaging in high-stakes gambling showed considerable individual differences (range: [−36, 25]; −2.9 ± 16.1), allowing us to evaluate its association with regional gray matter volume.

### 3.2. Propensity for Engaging in High-Stakes Gambling and vmPFC Volume

**Figure 3:**
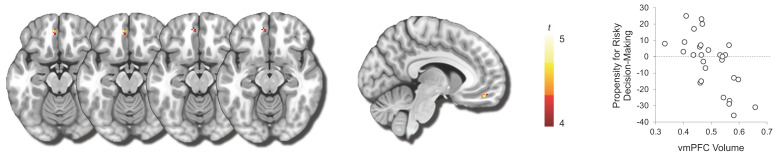
VBM results showing vmPFC voxels that showed negative correlation with the behavioral propensity for engaging in high-stakes gambling. Red–yellow areas highlight voxels that were significant using the TFCE method. Scatterplot is provided for visualization purposes only.

Gray matter volume in the vmPFC region was negatively correlated with the propensity for engaging in high-stakes gambling (MNI −8, 46, −16; *t*(28) = 4.89, FWE-corrected *p* < 0.05, k = 13 voxels) (Figure 3). Gray matter volume of this vmPFC cluster was not correlated with the overall tendency to participate in gambling activity (*r*(29) = 0.27, *p* = 0.16). No other regional gray matter volumes were either negatively or positively correlated with this behavioral index. Post hoc analysis showed that the mean gray-matter volume estimates of the other PFC regions (i.e., each of the PFC ROIs derived from the Harvard–Oxford cortical structural atlas) were not significantly correlated with the behavioral index after correcting for false discovery rate, supporting the spatial specificity of the main findings.

Control VBM analyses showed that neither the simple frequency of engaging in high-stakes gambling without adjusting for low-stakes gambling, nor the general tendency to engage in gambling behavior regardless of the stakes were associated with vmPFC/OFC volume.

## 4. Discussion

Here we present neuroimaging data highlighting a negative association between the volumetric variability of the vmPFC and the behavioral tendency to engage in high-stakes gambling. Expanding upon vmPFC/OFC lesion patient and pathological gambling studies, our findings suggest that individual differences in vmPFC structure are linked to normal variations in the propensity for seeking high-risk/high-reward situations.

Our experimental task design allowed us to examine the neural correlates of the behavioral tendency to engage in gambling behavior based on the level of potential gains and losses. Of note, in order to emulate real-world gambling scenarios, we introduced an additional step during which participants were given a choice to either accept or reject the gamble on a trial-by-trial basis. Based on the differences in the acceptance rates to high- vs. low-stakes gambling conditions, an individual differences metric reflecting the propensity to engage in high-stakes gambling was computed. Our analysis of structural brain data revealed that across the entire prefrontal region, only the vmPFC was identified as being associated with this behavioral index, demonstrating the spatial specificity of the observed effects.

The functional role of the vmPFC appears to be multiplexed, as it has been implicated in various psychological processes including valuation [24], decision-making in the absence of uncertainty [25], self–other representations [26], and moral judgment [27]. In the context of decision-making behavior, a large body of functional neuroimaging work has strongly suggested that the vmPFC encodes subjective value [1]. Indeed, converging evidence from functional MRI studies has demonstrated increased vmPFC activity corresponding to monetary reward [28,29] and subject-specific valuations of gains and losses [30]. Corroborating these findings from functional neuroimaging research, our study further demonstrates how individual differences in the behavioral responses to potential gains and losses mapped onto vmPFC structure.

Specifically, our data suggest that individuals with smaller vmPFC volume are more attracted to greater potential gains, and less aversive to greater potential losses. This observation is generally in line with previous studies showing that vmPFC/OFC lesion patients are less risk averse [3,31,32]. Based on the common finding that vmPFC/OFC lesion patients are more driven towards choices associated with greater potential gain, a possible interpretation of our data is that healthy individuals with smaller vmPFC are also more attracted to greater potential gain regardless of the risk involved. Another affective component at play here may be the emotion of regret in decision-making [33]. Based on prior work demonstrating that patients with vmPFC/OFC damage do not express regret in a decision-making context [34], individuals with smaller vmPFC might experience relatively less regret from the consequences of their choices. Perhaps these individuals are less influenced by better outcomes from a counterfactual action, especially when losses are involved (e.g., had they have not gambled, they would not have lost a large amount of money in the high-stakes gambling condition). While speculative, having fewer regrets about what could have been might allow them to simply focus on current choices with greater potential payoffs while discounting the risks. All that being said, of course, this does not directly imply that smaller vmPFC is a biomarker for pathological gambling behavior. In fact, the main contribution of our findings is that vmPFC volume may be sensitive to normal variations in the propensity to engage in risky gambling.

The present study is not without caveats that could be addressed in future research. The current findings are based on a relatively small sample largely composed of young adults, and thus would benefit from better-powered follow-up studies, especially considering that individual differences in structural variability may require very large samples for replication [35]. In addition, the methods used in the present study were unable to identify the exact neural mechanism underlying the link between vmPFC volume and gambling behavior. Considering the known functional role of the vmPFC in the representation of subjective value [1], perhaps smaller vmPFC may be indicative of altered value computation in the brain, leading to overvaluation of potential gain versus loss in a gambling context. Of course, such speculation must be made with caution, as the functional consequences of altered vmPFC volume, especially within the normal range, remain largely unknown.

These limitations notwithstanding, our data build upon and extend the well-documented findings from the vmPFC/OFC lesion patient studies. Instead of relying on self-report measures, we used a metric derived from each participant’s behavioral responses in a gambling task to show that individuals with smaller vmPFC were characterized by a greater propensity to engage in high-risk/high-reward gambling, which is likely to be driven by their affinity for greater potential gain over the risks posed by potential loss. By offering a possible neuroanatomical basis for risk-taking behavior in a value-based decision-making context [36], the present findings may be able to inform future translational research that aims to further elucidate the neural underpinnings of normative and pathological gambling.

## Data Availability

All VBM processed data and the behavioral index are publicly available at NeuroVault: https://neurovault.org/collections/13070/ (accessed on 20 October 2022).
